# Bioinformatics analysis suggests base modifications of tRNAs and miRNAs in *Arabidopsis thaliana*

**DOI:** 10.1186/1471-2164-10-155

**Published:** 2009-04-09

**Authors:** Kei Iida, Hailing Jin, Jian-Kang Zhu

**Affiliations:** 1 Department of Botany and Plant Sciences, University of California, Riverside, CA 92521, USA; 2 Bioinformatics and Systems Engineering Division, RIKEN Yokohama Institute, 1-7-22, Suehiro, Tsurumi, Yokohama 230-0045, Japan; 3 Department of Plant Pathology, University of California, Riverside, California 92521, USA

## Abstract

**Background:**

Modifications of RNA bases have been found in some mRNAs and non-coding RNAs including rRNAs, tRNAs, and snRNAs, where modified bases are important for RNA function. Little is known about RNA base modifications in *Arabidopsis thaliana*.

**Results:**

In the current work, we carried out a bioinformatics analysis of RNA base modifications in tRNAs and miRNAs using large numbers of cDNA sequences of small RNAs (sRNAs) generated with the 454 technology and the massively parallel signature sequencing (MPSS) method. We looked for sRNAs that map to the genome sequence with one-base mismatch (OMM), which indicate candidate modified nucleotides. We obtained 1,187 sites with possible RNA base modifications supported by both 454 and MPSS sequences. Seven hundred and three of these sites were within tRNA loci. Nucleotide substitutions were frequently located in the T arm (substitutions from A to U or G), upstream of the D arm (from G to C, U, or A), and downstream of the D arm (from G to U). The positions of major substitution sites corresponded with the following known RNA base modifications in tRNAs: N1-methyladenosine (m^1^A), N2-methylguanosine (m^2^G), and N2-N2-methylguanosine (m^2^_2_G).

**Conclusion:**

These results indicate that our bioinformatics method successfully detected modified nucleotides in tRNAs. Using this method, we also found 147 substitution sites in miRNA loci. As with tRNAs, substitutions from A to U or G and from G to C, U, or A were common, suggesting that base modifications might be similar in tRNAs and miRNAs. We suggest that miRNAs contain modified bases and such modifications might be important for miRNA maturation and/or function.

## Background

In rRNAs, tRNAs, snRNAs, and some mRNAs, the bases of nucleotides are often modified [1-5]. Especially in tRNAs, many types of base modifications have been characterized [1,2]. These modifications often involve methylation or acetylation, and may contribute to the stability of tRNA molecules when they form tertiary structures [4,6]. Another well-characterized tRNA modification is RNA editing from adenine (A) to inosine (I) [7,8]. This A to I editing is explained by deamination of A by adenosine deaminase [7]. In the yeast alanine tRNA, adenosine bases on the anticodon are deaminated to I. The edited inosine can form base pairs with uridine (U), cytosine (C), or adenosine in codons of mRNAs, and thereby expands the decoding capacities [8]. Although RNA modification in tRNAs, especially mitochondrial tRNAs, has been studied in *Arabidopsis *[9], the understanding of RNA modification in *Arabidopsis *tRNAs is still limited.

MicroRNAs (miRNAs) are a recently described class of functional RNAs and were initially found in *C. elegans *[10]. Several editing events in miRNAs have been reported. One example is A to I editing in primary miRNA precursor transcripts (pri-miRNAs). A to I editing has been documented for human pri-miR-22 and pri-miR-142 [11-13]. In the case of human pri-miR-142, A to I editing represses the maturation process carried out by the cleaving enzyme, Drosha [12,13]. Another example of RNA editing in miRNAs is found in primary transcripts of the human miR-376 cluster [14]. In this case, the edited pri-miRNAs are processed into mature miRNAs, and the mature miRNA targets different transcripts than the non-edited miRNA. These examples demonstrated the importance of RNA editing on miRNAs. Like humans, *Arabidopsis thaliana *also has hundreds of miRNA genes [15]. In *Arabidopsis*, the methyltransferase enzyme HEN1 introduces 2'-O methyl groups to the ribose of 3' terminal nucleotide of miRNAs [16,17]. This modification is important for the stability and accumulation of miRNAs. However, there has been no report of RNA modification in the bases of miRNAs in *Arabidopsis *or other plants.

The current study is focused on RNA base modifications in *Arabidopsis*. Because some modified bases are read differently from unmodified bases by reverse transcriptases during cDNA synthesis [18], we expect that the modified nucleotides will be read as different nucleotides from genomic ones if the bases of RNAs are modified. Our analysis utilized several sets of high throughput cDNA sequences of small RNAs (sRNAs). We used our own sequences generated by the "454" technology [19,20]. During the analysis of our sequences, we observed that many of the sequences could not be mapped to either the nuclear or organellar genomes [19]. We suspected that such non-mapped sequences may contain information of RNA modifications. We found that the sRNAs that were not mapped to the genome, mapped perfectly with the genome sequences except for one base mismatches (OMM). In the current work, we also used public sequences obtained with the 454 technology and the "massively parallel signature sequencing" (MPSS) method [21,22]. The MPSS dataset is especially important here since the sequences were generated by a completely different technology from the 454. We listed substituted sites only if the sites were supported by sequences from both 454 technology and MPSS. This strategy allowed us to avoid detecting simple sequencing errors resulting from one or the other of the sequencing technologies. In this study, we first analyzed RNA base modifications in tRNA molecules. Next, we investigated potential RNA base modifications in miRNAs. Ours is the first report of large scale analysis of RNA base modifications of tRNAs in *Arabidopsis*. More importantly, our bioinformatics analysis suggests similar base modifications (e.g. methylation and acetylation) in miRNAs.

## Results and discussion

### Mapping sRNAs to the genome

We mapped sRNA sequences to the *Arabidopsis *nuclear genome with the alignment program SOAP [23]. Out of 249,495 unique sRNA sequences, 121,319 (48.6%) were perfectly mapped to the genome. Another 32,220 sequences (12.9%) were mapped to the genome with one base mismatch (OMM). For the sRNAs obtained with the MPSS method, 7.7% of all sequence reads were OMM sRNAs, while 10.5% of public data [21] and 13.9% of our data obtained with the 454 technology were OMM sRNAs (Table 1). The error rate for each nucleotide has been estimated to be ~0.25% for MPSS sequences and 0.004% for 454 sequences [24,21]. If a sRNA was 18 nt long, which was the average in the current data sets, the percentage of sRNAs with OMM attributable to sequencing errors only was estimated to be 4.4% for MPSS sequences and 0.07% for 454 sequences. Observed rates of OMM sRNA were remarkably higher than these estimates based on sequencing errors, suggesting that OMM sRNAs were not due to simple sequencing errors. The lower rate of OMM sRNAs in the MPSS data set than in the 454 data set is probably caused by the shorter length of the MPSS sRNA sequences because short sequences with substitutions are more likely to have perfect matches to some genomic loci by chance.

**Table 1:** Data set and mapping results

	All	All	Perfect match	Perfect Match	OMM	OMM	OMM excluding terminal miss match	OMM excluding terminal miss match
	
	Unique	Reads	Unique	Reads	Unique	Reads	Unique	Reads
454 data set 1^a^	96,080	236,245	19,085 (19.9%)	69,071 (29.2%)	14,269 (14.9%)	32,808 (13.9%)	4,889 (5.1%)	11,162 (4.7%)
454 data set 2^b^	36,562	58,163	18,027 (49.3%)	18,027 (31.0%)	4,709 (12.9%)	6,081 (10.5%)	931 (2.5%)	1,091 (1.9%)
MPSS data set^c^	116,853	3,126,002	84,207 (72.1%)	2,501,573 (80.0%)	13,242 (11.3%)	240,355 (7.7%)	6,505 (5.6%)	112,299 (3.6%)

(a) Jin et al. 2008

(b) Lu et al. 2006

(c) Lu et al. 2005

### Finding candidate sRNAs with base modifications

**Figure 1:**
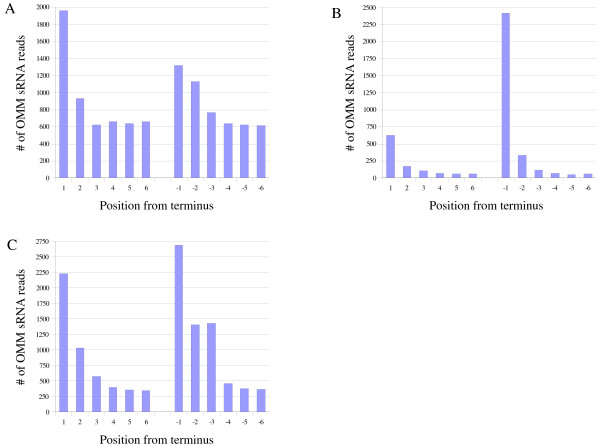
**Distribution of positions of observed substitutions in sRNA sequences**. Sequences were obtained with the MPSS method (A), with 454 technology by Lu et al. [22] (B), and our own sequence set (C). For each graph, the left side shows the number of substitutions associated with initial nucleotides (where "1" is the first nucleotide) and right side shows the number of substitutions associated with terminal nucleotides (where "-1" is the last nucleotide). For the entire data set, substitutions were especially abundant in the initial/terminal three nucleotides.

By examining the positions of mismatch sites of 32,220 OMM sRNAs, we determined that many mismatches occurred in the initial and terminal three bases (Fig. 1). Out of all 32,220 OMM sRNAs, 19,895 had their mismatch sites within the initial or terminal three bases. In sequences obtained with the 454 technology, previous research showed that accuracy was relatively low in the initial and terminal bases [20]. Thus, our further analysis did not use OMM sRNAs with mismatched nucleotides in the initial or terminal three bases. We applied the same criterion for the sequences obtained with MPSS technology, because we observed similar distributions of mismatched sites in sequences from both 454 and MPSS (Fig 1). Consequently, we selected 12,325 sRNA sequences for further analysis. Because one OMM sRNA may be mapped to multiple loci of the genome, 12,325 OMM sRNA sequences matched 34,010 loci with substitutions. We then selected loci that were supported by OMM sRNAs from both the 454 and MPSS data sets. Substitution sites were finally summarized into 1,187 sites in the genome sequences. Many of them (44%) were supported by two or three unique OMM sRNAs. We also found that a significant fraction (21%) of the loci were supported by more than 10 unique OMM sRNAs (see Additional file 1). We performed chi-square tests to determine how many of the sites had significantly greater numbers of OMM sRNAs than estimated numbers of sRNAs with sequencing errors. Out of 1,187 sites, 653 sites (55%) had statistical significances with *p*-values less than 0.01.

We also considered possibilities of single nucleotide polymorphisms (SNPs) contributing to the OMMs. In the case of SNPs, sequences from the same genetic background (such as Col-0) must have the same substitutions and should not have perfect matches. In our analysis, 1,166 sites out of all 1,187 substitution sites had mixtures of substituted and non-substituted nucleotides from plants of a single genetic background. Besides, in the remaining 21 sites, perfectly modified sites, such as A to I editing sites were included. These results suggest that OMMs did not come from SNPs.

We examined the genomic loci and classified the substitution sites based on the corresponding genome annotation. Out of 1,187 substitution sites, 703 sites corresponded with tRNAs (59.2%), 147 with miRNAs (12.4%), and 88 with ribosomal RNAs (7.4%).

### Base modifications in tRNAs

**Figure 2:**
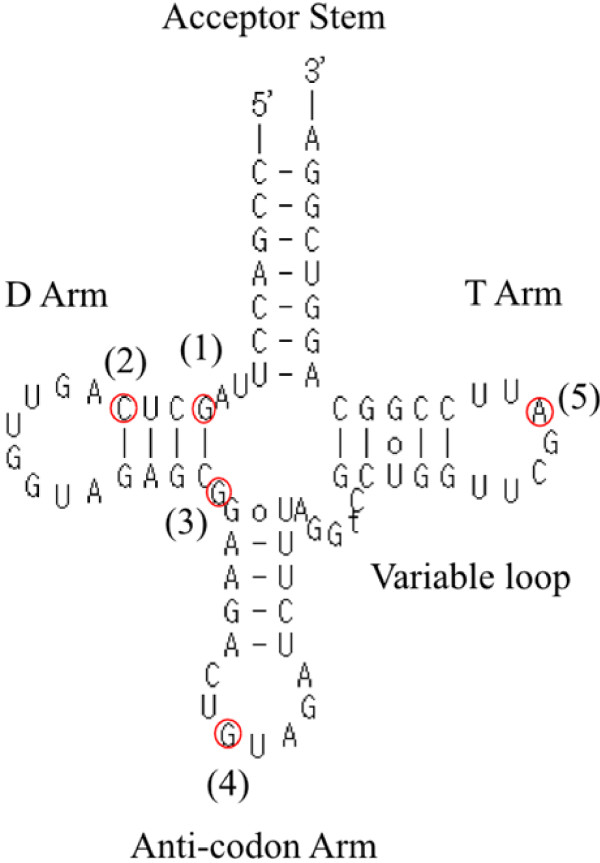
**An example of the clover leaf structure of tRNAs**. Shown is AT1G49460, which is the tRNA for tyrosine). Red circles indicate the positions of major RNA modification sites (Table 2). In this tRNA, substitutions were not found at positions (1), (4), or (5), but corresponding nucleotides are shown.

In the current analysis, the largest fraction of observed substitution sites corresponded with tRNAs. Because we used sRNAs of up to only 40 nt in length, we inferred that the tRNA sequences came from fragmented tRNAs. Such fragmented tRNAs would be consistent with recent reports of detection of cellular tRNA fragments [25,26]. Given that most substitution sites corresponded with tRNAs, these were the first substitution sites that we analyzed. We predicted secondary structures of tRNAs with tRNAscan-SE [27] and then mapped the substitution sites on the secondary structures. The substitution sites were clustered into five major regions and several minor regions (Table 2). The most abundant type of substitution was found in the T loop (Table 2, Fig. 2). In these regions, A to U or to G substitutions were common. Out of 639 tRNAs encoded in the *Arabidopsis *genome, 383 have substitutions of this type. We found this type of substitution on tRNAs for all amino acids except asparagine, methionine, and tyrosine. This position corresponds with the position of a known RNA modification, N1-methyladenosine (m^1^A) [5,28,29]. We concluded that the inferred modification was a methylation of adenine such that A was substituted with U or G in the cDNA sequences of the tRNA fragments.

**Table 2:** Modified nucleotides found in tRNA

**Region name**	**Genomic nucleotide**	**(a)**	**(b)**	**TPQ for non-modified bases**	**# of tRNA**	**Uniq # of sRNA**	**Total TPQ of sRNA**	**Edited nucleotides**	**TPQ of edited nucleotides**	**Type(s) of tRNA**
T_loop	A	(5)	m^1^A	4454.04	383	4198	12127.1	U,G,C	9005.67,2958.02,163.49	Ala,Arg,Asp,Cys,Gln,Glu,Gly,His,Ile,Leu,Lys,Phe,Pro,Ser,Thr,Trp,Val
D->AntiC	G	(3)	m^2^_2_G	372.31	34	257	894.15	U,A,C	660.14,155.40,78.64	Asn,Met,Tyr
Acc->D	G	(1)	m^2^G	23314.9	138	682	767.54	C,U,A	378.05,212.08,176.87	Arg,Lys,Met,Ser,Trp,Tyr,Val
D_stem_5'	C	(2)	ac^4^C	17339.82	17	51	212.86	A,U,G	197.48,8.42,6.96	Arg,Gly
AntiC_loop	A	(4)	I	0	6	86	98.95	G	98.95	Thr
D_stem_5'	T	N/A	N/A	11624.69	60	180	43.2	C,A	23.40,19.80	Tyr
Acc_stem_5'	G	N/A	N/A	16999.53	17	45	38.64	A,U	32.15,6.38	Arg,Gly
D_loop	G	N/A	N/A	2420.16	6	12	24.9	A	24.9	Ser
D_loop	A	N/A	N/A	1348	1	2	22	U,C	13.90,8.10	Gln
Acc_stem_5'	T	N/A	N/A	2406.42	6	12	11.4	C,G	9.42,1.98	Ser
AntiC_loop	G	N/A	m^1^G	47.12	26	52	9.1	U,C	8.32,0.78	Pro
T_stem_5'	G	N/A	N/A	313.29	3	6	8.61	A,C	7.89,0.69	Asp

(a) Corresponding position in Figure 2.

(b) Corresponding modified base.

The second most abundant type of substitutions were in the 3' regions of D loop (Table 2, Fig. 2). In these substitutions, genome encoded guanine (G) was read mainly as U. This position is known as the position of the modified nucleotide, N2-dimethylguanosine (also called N2-N2-methylguanosine or m^2^_2_G) [30-32]. Interestingly, this modification was found only in tRNAs for asparagine, methionine, and tyrosine, which did not have the m^1^A modification in the T loop. In the tertiary structure of tRNAs, m^1^A does not contact m^2^_2_G [33]. The relationship of these two types of modifications is unclear.

The third most abundant type of substitutions occurred between the D loop and acceptor stem, at the position of N2-methylguanosine (m^2^G) [5,6]. These substitutions may also involve modifications of guanine, as in m^2^_2_G, but the substitution pattern observed in cDNAs was different. We observed C, U, and A with similar abundance. We suggest that this difference in substitution patterns reflects the different effects of m^2^G and m^2^_2_G on reverse transcription, and may be used to predict m^2^G and m^2^_2_G modifications.

The fourth most abundant type of substitutions was found in the 5' part of the D stem, where A substituted for C (Table 2). This site corresponds to the known modification site of N4-acetylcytidine (ac^4^C) [4].

The fifth most abundant type of substitutions involved the well-known A to I editing in the anticodon loop (Table 1, ref. [7]). In these cases, adenine was read as G but not as U or C. This type of RNA editing was found in only six cases of threonine tRNA (Table 2). We noted that the editing rate in this case was 100% (Table 2). In the current analysis, we used sRNA sequences that included fragments of tRNAs. These tRNA fragments rarely contain the anticodon regions. Out of 5,657 OMM sRNAs supporting substitutions in tRNAs, only 274 (4.8%) contain anticodon nucleotides. These results indicate that most tRNA fragments were products of cleavage around anticodon regions, as is true for tRNA fragments found in humans or *Tetrahymena thermophila *[25,26]. This is probably one reason why few cases of A to I editing were observed in this study. Our elimination of OMM sRNAs with substitutions in the initial or terminal three bases might also have reduced the chance to observe modified bases on aniticodon loops. It is known that the anticodon loop has several modified nucleotides besides A to I editing [reviewed in ref. [34]]. Despite the small number of tRNA fragments containing the anticodon loop, we found some substitutions which correspond to the well-known modified base, 1-methylguanosine (m^1^G) at position 37 of the anticodon loop (Table 2, substitution of G to U/C).

The locations of predicted RNA modifications based on OMMs corresponded well with locations of known modifications reported in the literature. Out of 703 substitution sites found in tRNAs, 604 sites (86%) can be accounted for by the 6 known base modifications discussed above. Clearly these bias were not caused by chance, which was supported by binomial test with *p*-value less than 1*10^-15^. Substitution patterns were also consistent with the known chemistry of reverse transcription in the literature. Research on reverse transcription of tRNAs showed that m^1^A nucleotides formed base pairs with A, U, G, or C [18]. In agreement with Steinberg and Cedergren [31], we found that the substituted nucleotides at known m^2^_2_G sites were mainly U and A (Table 2). Therefore, our predictions of base modifications in tRNAs are consistent with previous studies. We suggest that RNA modification types can be classified based on substitution patterns found in OMM sRNAs.

### Base modifications in miRNAs

**Figure 3:**
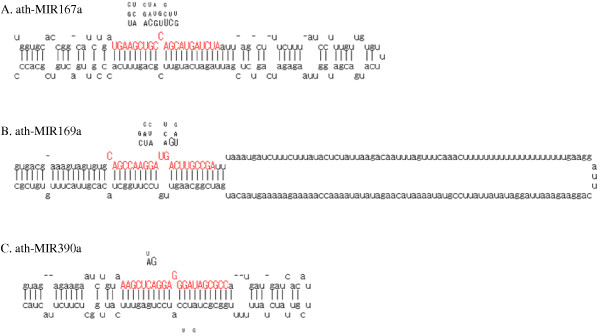
**Examples of substitutions found in miRNAs**. Red characters show sequences of mature miRNAs. Upper and lower characters show the position and nucleotides of substitutions. Size of the characters corresponds with the usage of the nucleotides.

**Figure 4:**
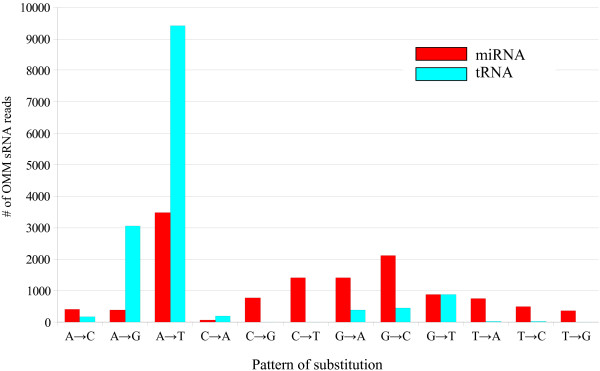
**Illustration of substitution patterns found in sRNAs corresponding to tRNAs**. Changes of A to T or G and G to T, C, or A are abundant. Similar substitution patterns were found in miRNAs.

After describing base modifications in tRNAs, we next considered possible base modifications in miRNAs. We found 147 cases of substitutions in miRNAs, which could be classified into 13 families (Fig. 3, Table 3). The substitution pattern of A was similar to that found in tRNAs (Fig 4). Adenine nucleotides were read mainly as thymine (T) and guanine in OMM sRNAs. This similarity of substitution suggests that N1-methyladenosines could be a major modified RNA base in miRNAs, as in tRNAs (Table 2). In the cases of substitutions of G, all of the other three nucleotides (C, A, and T) were observed (Fig 4). This substitution pattern was similar to that of m^2^_2_G rather than of m^2^G in tRNAs (Table 2). We suspect that m^2^_2_G might be the main modified G in miRNAs, although it is possible that miRNAs may have different types of G modifications. We also examined the substitutions on genomic C and T sites, although there are few such substitutions in tRNAs. These RNA modifications may differ from those in tRNAs or may be rare in tRNAs. We found that some fraction of the substitutions occurring in miRNAs were similar with those in tRNAs. Moreover, the substitution patterns were not random. Based on these results, we concluded that these substitutions are not simple sequencing errors, but reflect novel RNA base modifications.

**Table 3:** Substitutions found in miRNAs

**Name of microRNA**	**Summary of substitutions^a^**
MIR159a, MIR159b	5:G(27900.95)>CA(21), 12:G(28142.15)>CA(45.4), 13:G(35092.7)>UA(144.86)

MIR161	6:G(20643.65)>CA(50.2), 9:A(20057.15)>GCU(109.2), 13:C(18208.1)>GA(8.3)

MIR162a, MIR162b	14:G(3040.5)>UA(58.8)

MIR165a, MIR166a, MIR166b, MIR166c, MIR166d, MIR166e, MIR166f, MIR166g	4:G(54554.7)>CUA(134.7), 6:C(54554.7)>UA(209.21), 7:C(44547.6)>U(149.93), 8:A(35038.15)>GU(83.7), 9:G(47675.1)>CUA(328.62), 10:G(54539.9)>A(106.29), 11:C(54539.9)>GUA(278.15), 12:U(54539.9)>GCA(379.38), 13:U(54539.9)>CA(256.11)

MIR167a, MIR167b, MIR167d	4:A(219813.04)>GCU(76.99), 5:G(219813.04)>CUA(325.81), 7:U(158835.06)>GCA(466.4), 8:G(158835.06)>CUA(326.2), 9:C(158835.06)>GUA(602.6), 10:C(158835.06)>GU(431.4), 11:A(219826.94)>GCU(3686.02), 12:G(219826.94)>CU(1018.4), 13:C(158835.06)>GU(336.74)

MIR168a, MIR168b	9:U(15948.36)>CA(36.6), 10:G(15933.26)>A(24.3), 12:A(15933.26)>GC(22.2), 13:G(15933.26)>UA(43.1), 14:G(15933.26)>UA(41.3)

MIR169a, MIR169b, MIR169c, MIR169d, MIR169e, MIR169h, MIR169i, MIR169j, MIR169k, MIR169l, MIR169m, MIR169n	6:C(24472.01)>GU(40.32), 8:A(87930.11)>GCU(77.31), 9:G(58812.37)>CUA(501.61), 10:G(37156.57)>CUA(161.5), 11:A(10397.81)>GC(26.88), 13:G(87930.11)>CUA(1043.6), 14:A(34341.26)>G(44.23), 15:C(38019.92)>GUA(109.1)

MIR170	5:U(50752.3)>GC(244.7), 8:G(50752.3)>CA(23.8)

MIR171a	7:A(2397.55)>GU(54.5)

MIR172a, MIR172b, MIR172c	8:U(211955.28)>GC(157.21), 11:U(83771.26)>C(90.98)

MIR319a, MIR319b	9:A(1866.44)>CU(37)

MIR390a, MIR390b	PRE74:A(6807.15)>G(21.5), PRE76:C(6807.15)>U(78.6), 6:C(41119.9)>UA(27.6), 7:A(20563.45)>G(48.5)

MIR858	5:G(12416.25)>CU(13.15), 12:G(12416.25)>CA(8.55)

(a) Format here is: position in mature miRNA:un-substituted nucleotide at this position (TQP of miRNAs without substitution) > substituted nucleotide(s) (TQP of miRNAs with substitution). In MIR390, two substitutions were found in pre-miRNA regions; the positions and frequncies of these subsitutions are indicated following "PRE": position from 5' end of pre-miRNA:unsubstituted nucleotide at this position (TPQ of sRNAs without substitution)>substituted nucleotide (TPQ of the sRNAs with substitution).

A to I editing is found in human miRNAs [14]. Based on the results with tRNAs, A to I editing was expected to be observed as substitution to G (Table 2). Such substitutions were found in miR169 (site 14) and miR319 (site 7) (Table 3). However, A to G substitutions did not represent the major substitution pattern (Fig. 4). Therefore, although A to I editing seems to occur in *Arabidopsis *miRNAs, such modifications are evidently uncommon.

### Potential biological impacts of base modifications in miRNAs

Our bioinformatics analysis strongly suggested RNA base modifications in miRNAs. What would be the biological effects of base modifications in miRNAs? We considered two possibilities. The first one is that the modification may enhance or prohibit the recognition of mRNA targets or cause recognition of novel targets, as is the case for RNA editing in human miR-376 [14]. The other possibility is that RNA base modifications may affect the maturation of miRNA, as with RNA editing in human pri-miR-142 [12,13].

**Figure 5:**
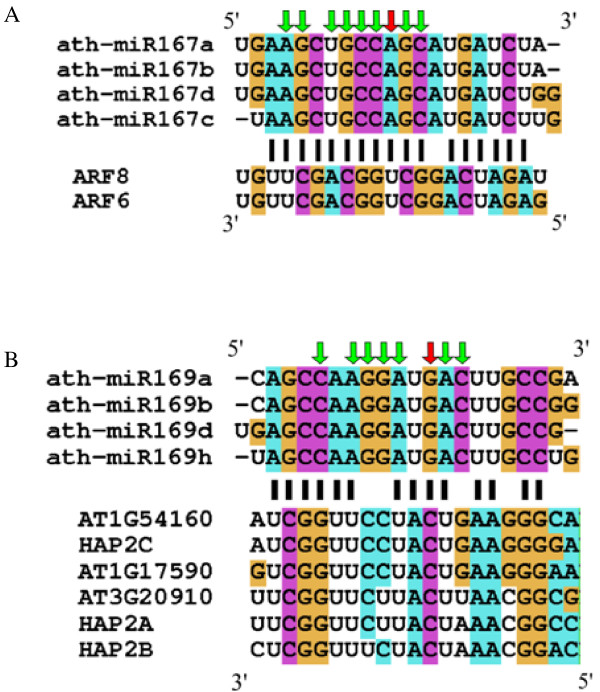
**Examples of substitutions found in miRNAs and relationship with their targets**. We show miRNAs that have the most (miR167; A) and the second most abundant (miR169; B) substitutions. In both cases, complementarity between miRNAs and their targets was reduced by the substitutions.

With respect to the first hypothesis, we examined how putative RNA modification sites may affect the relationship between miRNAs and their targets, assuming that the pairing between a modified nucleotide and its target nucleotide can be mimicked by pairing of the substituted nucleotide and the target (Fig. 5). Although the RNA modifications occurred in sites that could alter target recognition, the modifications did not seem to enhance target recognition. We also tried to predict some novel target transcripts with modified miRNA sequences but we were unable to find novel targets with miRNAs containing substitutions. These results suggest that the base modifications may not enhance target recognition or create new targets. Consistent with this notion, putative modification sites are spread widely within the length of a miRNA molecule (Fig. 3).

**Figure 6:**
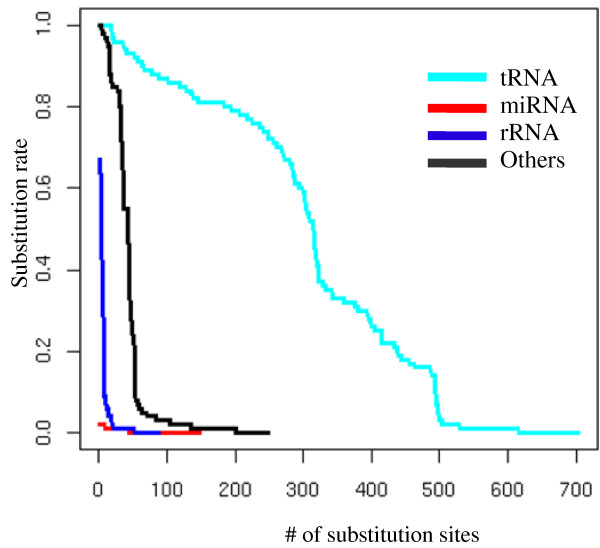
**The rates of substitutions for tRNAs, miRNAs, rRNAs, and all other data**. In tRNAs, substitution rates were more than 0.2 at 438 sites. In miRNAs, however, substitution rate was only 0.02 at the site with maximal substitution rate.

The second hypothesis was difficult to test because our data came from mature miRNAs rather than pri-miRNA sequences. However, several results in this study may be consistent with the second hypothesis. First, we found that nucleotides outside of mature miRNAs were also modified in miR390a (Fig. 3). Second, we surveyed EST sequences corresponding with pre-miRNAs. They also had several substitutions from genomic nucleotides that were located outside of regions of mature miRNAs (see Additional files 2 and 3). These results suggest that pre-miRNA already have modified bases. Third, it is known that several RNA modification enzymes affecting tRNAs recognize secondary and/or tertiary structures of tRNA molecules [35-37]. It is possible that tRNA modification enzymes might also modify pri-/pre miRNAs. In the case of miRNAs, it is unknown whether the base modifications may reinforce the fold-back structures of pre-miRNAs. However, we observed that many substitutions were in the positions where Watson-Crick or wobble base pairs were initially observed (Fig. 3). In such cases, the modifications may prevent the formation of the fold-back structures. Thus, it is possible that modified pri-/pre- miRNA are less efficiently recognized than non-modified ones by the processing enzyme, DCL1. The low modification rate (Table 3, Fig. 6) in the current data sets is also consistent with the second hypothesis, which proposes that modified pri/pre miRNAs are more prone to degradation and thus less likely to yield mature miRNAs [12,13]. Our analysis suggests that base modifications in miRNAs may prevent the maturation of miRNAs, although it is also possible that some base modifications in miRNAs may inhibit or enhance the targeting of miRNAs.

## Conclusion

Our data on tRNAs clearly show that substitutions in cDNAs can indicate RNA base modifications. This research thus found a new analytical strategy for predicting RNA base modifications and provided the first detailed bioinformatics analysis on RNA modifications in *Arabidopsis *tRNAs.

More importantly, this is the first report of the possibility of RNA base modifications in miRNAs with methyl or acetyl groups. Our results, which are based on carefully chosen sRNA sequences, strongly suggest that some RNA bases might be modified in miRNAs. We showed that one-base mismatches in sRNAs were not random and much more abundant than expected, and that many substitution sites were supported by both MPSS and 454 technologies. It follows that these substitutions cannot be explained by sequencing errors. More importantly, substitution patterns in adenine and guanine of miRNAs were similar to those of tRNAs. This similarity suggests common base modifications in miRNAs and tRNAs, although the nature of base modifications in miRNAs can only be determined experimentally in the future. Base modifications likely provide another regulatory mechanism for the biogenesis and/or functioning of miRNAs.

## Methods

### Data sets

We used 238,538 sRNA sequences (of which 98,035 were unique) sequenced by the 454 technology (Table 1) [19,20]. The small RNA libraries were constructed from plants treated withabiotic stresses (cold, drought, salt, copper, UV and ABA, respectively) or infected with bacterial or fungal pathogens [38]. We also used public sRNA sequences obtained by Lu et al. [21,22] with 454 and MPSS technologies, which have 58,178 reads and 3,126,002 reads respectively. The read numbers are used with normalization in TPQ (Transcripts Per Quarter million), according to ref. [21]. When a single type of sRNA had multiple loci on the genome, the TPQ value was divided by the number of the loci.

### Mapping sRNAs to the genome

We mapped sRNA sequences to the genome sequences with the alignment program, SOAP [23]. We used the program with the "-v 1" option, enabling the program to find one mismatch when the sRNA is mapped to the genome. We added a further criterion for the sRNAs that mapped to the genome with one mismatch. If a single sRNA had more than two OMM loci, with substitutions in different positions or with different nucleotides, we discarded it. After the analysis of the substitution sites (Table 1, Fig 1), we selected OMM sRNAs only if the substitution sites were not in the initial or terminal three bases. Then, we compared the results of sRNAs from 454 and MPSS technologies. If a substitution of a genomic locus was supported with sRNAs from both 454 and MPSS technologies, we selected it for further analysis. These criteria decreased technology-dependent errors.

### Statistical tests

We used probabilities of OMM sRNAs caused by sequencing errors based on reported sequencing error rates [24,21]: 0.25% for MPSS sequences and 0.004% for 454 sequences. We calculated the expected numbers of OMM sRNAs caused by sequencing errors for each substitution site. Then we performed chi-square tests to assess whether the read numbers of OMM sRNAs were significantly larger than expected from sequencing errors.

### Analysis of tRNAs and miRNAs

In the analysis of RNA modification in tRNAs and miRNAs, we had to determine the clover leaf structure of tRNAs and fold-back structures of miRNAs. For this purpose, we used tRNAscan-SE [27] and information about miRNAs published at miRBase [39]. Perl scripts were used to summarize the results.

## Authors' contributions

KI performed all bioinformatics analyses. HJ contributed sRNA sequences. JKZ conceived of the study. KI and JKZ wrote the paper. All authors read and approved the final manuscript.

## Supplementary Material

Additional File 1 **Distribution of numbers of OMM sRNAs supporting substitutions**. 249 of total 1,187 sites (21.0%) were supported by the minimal number of unique OMM sRNAs (one OMM sRNA from 454 data set and one from MPSS data set). This graph has a local peak where the number of unique OMM sRNA is six. 245 substitution sites were supported more than 10 unique OMM sRNAs. This graph shows the numbers of unique OMM sRNAs supporting each substitution site. Click here for file

Additional File 2 **Substitutions found in EST sequences for pre-miRNAs**. Panels A, B, and C show multiple alignments of non-substituted sequences and EST sequences for ath-MIR398c, ath-MIR835, and ath-MIR860, respectively. In the multiple alignments lines labeled with miRNA IDs represent non-substituted sequences. In these lines, nucleotides included in mature miRNAs are shown as capital letters. Bottom lines of each alignment block indicate the positions of substitutions. Note that substitution sites were observed not only within mature miRNA regions, but also outside of the mature miRNAs. Pound symbols in panel C indicate the region outside of EST. Click here for file

Additional File 3 **Detail of substitutions**. Raw information of all observed base modifications was described in the file. The information was summarized according to categories of corresponding genes (tRNA, miRNA, rRNA, and the others). Genomic positions of the observed base modifications, bases of nucleotides in the cases of unsubstituted and substituted, TPQs supporting each bases, definitions for corresponding genes, and results of chi-squared tests are described. Details of the chi-squared test were described in methods. Click here for file
