# Supplementary material for: Bioinformatics analysis suggests base modifications of tRNAs and miRNAs in Arabidopsis thaliana
Source: BMC Genomics. 2009 Apr 9;10:155. doi: 10.1186/1471-2164-10-155 (PMC2674459; doi:10.1186/1471-2164-10-155)
Supplement: Additional File 1 — Distribution of numbers of OMM sRNAs supporting substitutions. 249 of total 1,187 sites (21.0%) were supported by the minimal number of unique OMM sRNAs (one OMM sRNA from 454 data set and one from MPSS data set). This graph has a local peak where the number of unique OMM sRNA is six. 245 substitution sites were supported more than 10 unique OMM sRNAs. This graph shows the numbers of unique OMM sRNAs supporting each substitution site. [file 1471-2164-10-155-S1.ppt]

## Slide 1
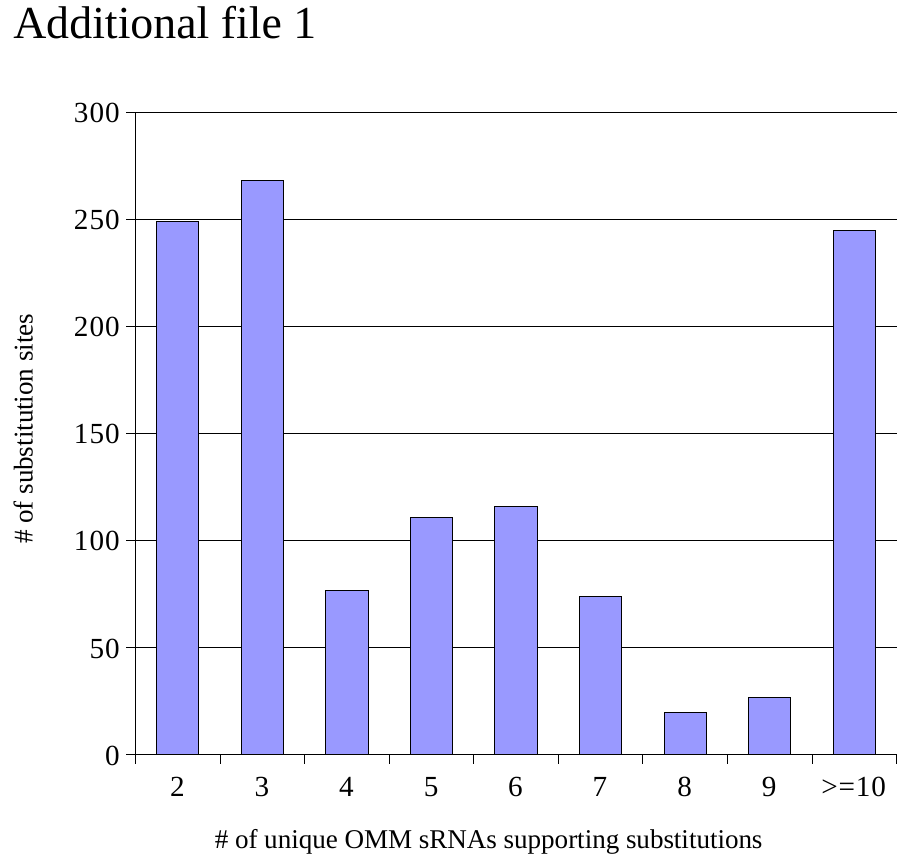

Additional file 1
### Chart
| Category | 列 E |
|---|---|
| 2 | 249.0 |
| 3 | 268.0 |
| 4 | 77.0 |
| 5 | 111.0 |
| 6 | 116.0 |
| 7 | 74.0 |
| 8 | 20.0 |
| 9 | 27.0 |
| >=10 | 245.0 |# of substitution sites
# of unique OMM sRNAs supporting substitutions
