# Supplementary material for: Bioinformatics analysis suggests base modifications of tRNAs and miRNAs in Arabidopsis thaliana
Source: BMC Genomics. 2009 Apr 9;10:155. doi: 10.1186/1471-2164-10-155 (PMC2674459; doi:10.1186/1471-2164-10-155)
Supplement: Additional File 2 — Substitutions found in EST sequences for pre-miRNAs. Panels A, B, and C show multiple alignments of non-substituted sequences and EST sequences for ath-MIR398c, ath-MIR835, and ath-MIR860, respectively. In the multiple alignments lines labeled with miRNA IDs represent non-substituted sequences. In these lines, nucleotides included in mature miRNAs are shown as capital letters. Bottom lines of each alignment block indicate the positions of substitutions. Note that substitution sites were observed not only within mature miRNA regions, but also outside of the mature miRNAs. Pound symbols in panel C indicate the region outside of EST. [file 1471-2164-10-155-S2.pdf]

A. ath-MIR398c

```
DR359082         TGGATCTCGACAGGGTTGATATGAGAACACACGAGCAATCAACGGCTATAACGACGCTAC
DR359083         TGGATCTCGACAGGGTTGATATGAGAACACACGAGCAATCAACGGCTATAACGACGCTAC
ath-MIR398c      tggatctcgacagggttgatatgagaacacacgagcaatcaacggctataacgacgctac
Substitution

DR359082         GTCATTGTTACAGCTCTCATTTCATGTGTTCTCAGGTCACCCCTGCTGAGCTCTT
DR359083         GTCATTGTTACAGCTCTCATTTCAAGTGTTCTCAGGTCACCCCTGCTGAGCTCTT
ath-MIR398c      gtcattgttacagctctcgtttcaTGTGTTCTCAGGTCACCCCTGctgagctctt
Substitution                       *     *
```

B. ath-MIR835

```
BX818094         TGCTCGATTCTTCAGAAAGAAATTCTGGTCATCTTTGTGTTTAGGCTTTTTCTTGCATGT
EG450195         TGCTCGATTCTTCAGATAGAAATTCTGGTCATCTTTGTGTTTAGGCTTTTTCTTGCATAT
EG476863         TGCTCGATTCTTCAGATAGAAATTCTGGTCATCTTTGTGTTTAGGCTTTTTCTTGCATAT
EG476865         TGCTCGATTCTTCAGATAGAAATTCTGGTCATCTTTGTGTTTAGGCTTTTTCTTGCATAT
EF183269         TGCTCGATTCTTCAGATAGAAATTCTGGTCATCTTTGTGTTTAGGCTTTTTCTTGCATAT
ath-MIR835       tgctcgattcttcagatagaaattctggtcatctttgtgtttaggctttTTCTTGCATAT
Substitution                     *                                         *

BX818094         GTTCTTTATCTCTATTGATTGATATTGTTCAATGAGCAACATACTTTTCTCTTCTACCTG
EG450195         GTTCTTTATCTCTATTGATTGATATTGTTCAATGAGCAACATACTTTTTTTTTTTACCTG
EG476863         GTTCTTTATCTCTATTGATTGATATTGTTCAATGAGCAACATACTTTTCTCTTCTACCTG
EG476865         GTTCTTTATCTCTATTGATTGATATTGTTCAATGAGCAACATACTTTTCTCTTCTACCTG
EF183269         GTTCTTTATCTCTATTGATTGATATTGTTCAATGAGCAACATACTTTTCTCTTCTACCTG
ath-MIR835       GTTCTTTATCtctattgattgatattgttcaatgagcaacatacttttctcttctacctg
Substitution                                                     * *  *

BX818094         GATTTGTTTGATGGTTTTAAGAACTCTGTTCTTTTTGACGTTTTACCCAACAATATAACT
EG450195         GATTTGTTTGATGGTTTTAAGAACTCTGTTCTTTTTGACGTTTTACCCAACAATATAACT
EG476863         GATTTGTTTGATGGTTTTAAGAACTCTGTTCTTTTTGACGTTTTACCCAACAATATAACT
EG476865         GATTTGTTTGATGGTTTTAAGAACTCTGTTCTTTTTGACGTTTTACCCAACAATATAACT
EF183269         GATTTGTTTGATGGTTTTAAGAACTCTGTTCTTTTTGACGTTTTACCCAACAATATAACT
ath-MIR835       gatttgtttgatggttttaagaactctgttctttttgacgttttacccaacaatataact
Substitution

BX818094         TCTGTTGTAGTCATCTTCTGCTGCAACCCATCCACAAAGCTTTTCATGCAAGATTAAACC
EG450195         TCTGTTGTAGTCATCTTTTGCTGCAACCCATCCACAAAGCTTATCATGCAAGATTAAACC
EG476863         TCTGTTGTAGTCATCTTTTGCTGCAACCCATCCACAAAGCTTATCATGCAAGATTAAACC
EG476865         TCTGTTGTAGTCATCTTCTGCTGCAACCCATCCACAAAGCTTATCATGCAAGATTAAACC
EF183269         TCTGTTGTAGTCATCTTCTGCTGCAACCCATCCACAAAGCTTATCATGCAAGATTAAACC
ath-MIR835       tctgttgtagtcatcttctgctgcaacccatccacaaagcttatcatgcaagattaaacc
Substitution                      *                        *

BX818094         CTTTCTCGATGGGTTGCAAGAGAACATAACTAAAATAGAAGTAACATTGTTGGGGAAAAA
EG450195         CTTTCTCGATGGGTTGCAAGAGAACATCACTAAAATAGAAGTAACATTGTTGGGGAAAAA
EG476863         CTTTCTCGATGGGTTGCAAGAGAACATCACTAAAATAGAAGTAACATTGTTGGGGAAAAA
EG476865         CTTTCTCGATGGGTTGCAAGAGAACATCACTAAAATAGAAGTAACATTGTTGGGGAAAAA
EF183269         CTTTCTCGATGGGTTGCAAGAGAACATCACTAAAATAGAAGTAACATTGTTGGGGAAAAA
ath-MIR835       ctttctcgatgggttgcaagagaacatcactaaaatagaagtaacattgttggggaaaaa
Substitution                                *
```

```
BX818094        CGTCACAAATCCAGGTGTTAGAGAATAGGATGCTGATCGTTGAAAATTACTATCTATAAA
EG450195        CGTCACGAATCCAGGTATTAGAGAATAGGATGCTGATCGTTGAAAATAACTATCTATAAA
EG476863        CGTCACGAATCCAGGTATTAGAGAATAGGATGCTGATCGTTGAAAATAACTATCTATAAA
EG476865        CGTCACGAATCCAGGTATTAGAGAATAGGATGCTGATCGTTGAAAATAACTATCTATAAA
EF183269        CGTCACGAATCCAGGTATTAGAGAATAGGATGCTGATCGTTGAAAATAACTATCTATAAA
ath-MIR835      cgtcacaaatccaggtattagagaataggatgctgatcgttgaaaataactatctataaa
Substitution          *         *                              *

BX818094        GATGGAGAAGATACGCAAGAAAGAGTTGTATACACAAAGC
EG450195        GATGGAGAAGATACGCAAGAAAGAGTTGTAAACACAAAGC
EG476863        GATGGAGAAGATACGCAAGAAAGAGTTGTAAACACAAAGC
EG476865        GATGGAGAAGATACGCAAGAAAGAGTTGTAAACACAAAGC
EF183269        GATGGAGAAGATACGCAAGAAAGAGTTGTAAACACAAAGC
ath-MIR835      gaTGGAGAAGATACGCAAGAAAGagttgtaaacacaaagc
Substitution                                  *
```

C. ath-MIR860

```
AV785609        ####CTTGGGGA-ATTCAAGATATATAGTCCAATCTATTGAAGTACTAGTACACCAGCTC
BP647171        ATGTCTTGGTTAAATTCAATATATATAGTCCCATCTATTGAAGTACTAGTACACCAGCTC
BP596532        ATGTCTTGGTTAAATTCAATATATATAGTCCCATCTATTGAAGTACTAGTACACCAGCTC
AV439790        ATGTCTTGGTTAAATTCAATATATATAGTCCAATCTATTGAAGTACTAGTACGCCAGCTC
BP638182        ATGTCTTGGTTAAATTCAATATATATAGTCCAATCTATTGAAGTACTAGTACACCACCTC
AV542165        ATGTCTTGGTTAAATTCAATATATATAGTCCAATCTATTGAAGTACTAGTACACCAGCTC
AV544231        ATGTCTTGGTTAAATTCAATATATATAGTCCAATCTATTGAAGTACTAGTACACCAGCTC
ath-MIR860      atgtcttggttaaattcaatatatatagtccaatctattgaagtactagtacaccagctc
Substitution             **        *           *                    *   *

AV785609        TAATAAGCTGATGTGGGTAAGTAGTTCAATAGATT-GACTATGTATATTAAATTTGACTA
BP647171        TAATAAGCTGATGTGGGTAAGTAGTTCAATAGATTGGACTATGTATATTAAATTTGACTA
BP596532        TAATAAGCTGATGTGGGTAAGTAGTTCAATAGATTGGACTATGTATATTAAATTTGACTA
AV439790        TAATAAGCTGATGTGGGTAAGTAGTTCAATAGATTGGACTATGTATATTAAAGTTGACTA
BP638182        TAATAAGCTGATGTGGGTAAGTAGTTCAATAGATTGGACTATGTATATTAAATTTGACTA
AV542165        TAATAAGCTGATCTGGGTAAGTAGTTCACTAGATTGGACTATGTATATTAAATTTGCCTA
AV544231        TAATAAGCTGATGTGGGTAAATAGTTCAATAGATTGGACTATGTATATTAAATTTGACTA
ath-MIR860      taataagctgatgtgggtaagtagtTCAATAGATTGGACTATGTATattaaatttgacta
Substitution                *       *       *                       *   *

AV785609        AGAGAT
BP647171        AGAGAT
BP596532        AGAGAT
AV439790        AGAGAT
BP638182        AGAGAT
AV542165        AGAGCT
AV544231        AGAGAT
ath-MIR860      agagat
Substitution        *
```
